# Genome-Wide Identification of the *ARF* Gene Family and ARF3 Target Genes Regulating Ovary Initiation in Hazel via ChIP Sequencing

**DOI:** 10.3389/fpls.2021.715820

**Published:** 2021-08-10

**Authors:** Heng Wei, Yunqing Cheng, Ying Sun, Xingzheng Zhang, Hongli He, Jianfeng Liu

**Affiliations:** Jilin Provincial Key Laboratory of Plant Resource Science and Green Production, Jilin Normal University, Siping, China

**Keywords:** hazel, auxin response factor, ovary initiation, ovule development, ChIP-Seq

## Abstract

Hazel (*Corylus* spp.) is an economically important nut species with a unique biological characteristic of ovary differentiation and development initiating from the ovary primordium after pollination. Auxin participates in ovary initiation and has an essential impact on hazel fruit yield and quality. The regulation of auxin in ovary development is thought to be related to auxin response factors (ARFs); however, its detailed regulatory mechanism remains unclear. The spatiotemporal expression pattern of *C. heterophylla* ARF3 (ChARF3) was accessed via *ARF* gene family member identification and expression abundance analysis as well as immunohistochemistry. ChARF3 target genes were identified via chromatin immunoprecipitation followed by next-generation sequencing (ChIP-Seq). In total, 14 *ChARF* members containing at least B3 and Auxin_resp domains were found to be distributed on 9 of 11 chromosomes, and the protein molecular weights were predicted to range from 70.93–139.22 kD. Among eight differentially expressed *ChARFs, ChARF3* showed the most significant differences over four ovary developmental stages. Immunohistochemical analysis revealed that ChARF3 was expressed in the ovary primordium and funiculus, integument, endosperm, radicle, and cotyledon indicating its potential regulatory roles in ovary differentiation and development. In total, 3,167 ChARF3 target genes were identified through ChIP-Seq in four ovary developmental stages and were significantly enriched in the biosynthesis of secondary metabolites (ko01110), phenylpropanoid biosynthesis (ko00940), and phytohormone signal transduction (ko04075). ChARF3 was hypothesized to be involved in the regulation of auxin-induced genes and the transcription factors *MADS, AP2/ERF, TCP, FT*, and *LFY*. These results suggest that ChARF3 may regulate ovary initiation and ovule development by mediating genes related to auxin biosynthesis and transport, cell division and proliferation, and flower and fruit development. This study provides new insights into the molecular mechanism of hazel yield formation.

## Introduction

Hazel (*Corylus* spp.) is an economically important nut species belonging to the family Betulaceae. Hazel kernel is a traditional dry food and an essential raw material used for oil, powder, jam, and kernel crumb in food processing industries (Amaral et al., 2006). The final fruit size and kernel plumpness, the most desirable traits determining the hazelnut market price, are determined by ovary initiation and ovule filling, which comprise a complex series of developmental events (Cheng et al., 2018b; Liu et al., 2020). Fruit initiation (namely fruit set), an important reproductive process, is defined as the development of an ovary into a fast-growing young fruit via successful pollination and fertilization (Tang et al., 2015). In most flowering plants, the ovule containing the egg cells is present at the time of pollination, resulting in a short fertilization window (Liu et al., 2014a). However, in hazel, the entire ovary and ovules are absent at the time of pollination (Liu et al., 2014a); the ovary and ovule primordium begin to differentiate only after pollination and extension of the pollen tube to the stigma and are gradually formed within ~50 days, resulting in a much longer window for fertilization of the ovule and embryo sac maturation (Liu et al., 2014a,b). There are only several layers of ovary primordium cells in the female inflorescence at the time of blooming, and the ovary comprises pericarp, parenchyma, and ovule when fertilization is completed (Liu et al., 2014a,b). During this period, the ovary undergoes a series of complex biological events, and a better understanding of the initiation of ovary development is beneficial to clarify the mechanism of hazel yield formation.

Auxin concentration in the female inflorescence is promoted by pollination, and auxin tends to accumulate at the growth center of pistillate inflorescences and young ovaries, suggesting its important role in regulating ovary differentiation and development (Cheng et al., 2018b). Auxin response factors (ARFs) play central roles in conferring auxin-mediated responses by selecting target genes in plants (Liscum and Reed, 2002). At present, knowledge of the biological function of individual ARFs related to flower and fruit development has been mainly obtained from the studies of model plants, such as *Arabidopsis thaliana* (Li et al., 2016b). In *A. thaliana*, ARF1 and ARF2 regulate senescence and floral organ abscission (Ellis et al., 2005). Functional analysis of ARF2 indicates that it regulates auxin signaling, cell division, and the size of seeds and other organs (Ellis et al., 2005). ARF3/ETTIN (ETT) interacts with AGAMOUS (AG) and APETALA2 (AP2) in floral meristem determinacy (Liu et al., 2014). ARF5/MONOPTEROS (MP) is critically required for embryonic root and ovule development (Weijers et al., 2006; Cucinotta et al., 2021). ARF8 regulates fertilization and fruit development (Goetz et al., 2006), whereas ARF6 and ARF8 act redundantly in flower maturation (Nagpal et al., 2005). Previous studies have confirmed the important role of ARFs in the regulation of flower and fruit development. In this study, all members of the *ARF* gene family in the hazel genome were identified, followed by the identification of *C. heterophylla ARF* (*ChARF*) at the transcriptional level. Furthermore, immunohistochemical (IHC) analysis using ChARF3-specific antibodies was performed to provide molecular evidence of the involvement of ChARF3 in ovary development. Finally, ChARF3 target genes were identified via chromatin immunoprecipitation followed by next-generation sequencing (ChIP-Seq). This study provides new insights into the molecular mechanism of hazel yield formation.

## Materials and Methods

### Plant Materials

In 2019, plant samples were collected from a hazel orchard in Siping, Jilin Province, China. The primary hazelnut cultivar was *C. heterophylla* × *C. avellana* “Dawei” using *C. heterophylla* × *C. avellana* “Bokehong” as a pollination cultivar. These cultivars were identified using a simple sequence repeat-based technique with seven primer pairs (Cheng et al., 2018a) at the College of Life Sciences, Jilin Normal University. Sampling and sample pretreatment for the IHC and ChIP experiments were performed as described in previous studies (Cheng et al., 2018b; Liu et al., 2020), with minor modifications. In total, 40 15-year-old “Dawei” trees were randomly selected and used as the study material. On April 7, ~2,000 quality “Dawei” pistillate inflorescences were randomly bagged and tagged; each of the selected trees had approximately 50 tagged inflorescences. On April 17, more than 18 0.5-m-long twigs were cut from six study trees for exogenous auxin and auxin inhibitor treatment. On April 20 (blooming date), artificial pollination was performed to exclude the possibility of self-pollination on the same day. For further IHC and ChIP experiments, samples were collected on May 30, June 30, and July 30, 10 days later than that described before and between the sampling time points of ovule formation (Ov1 stage), early ovule growth (Ov2 stage), rapid ovule growth (Ov3 stage), and ovule maturity (Ov4 stage), respectively. Thus, the samples used in this study were named Ov1.1, Ov2.1, and Ov3.1 to distinguish them from previous samples for RNA sequencing (RNA-Seq) (Liu et al., 2020). For the IHC experiment, pistillate inflorescences or young fruit were immediately placed in liquid nitrogen. For the ChIP experiment, ovules [>0.1 g (g fresh weight)] were isolated manually from the pistillate inflorescences or fruit clusters; thereafter, only medium-sized ovules, among all isolated ovules from the same development stage, were immediately treated with 1% formaldehyde before placing in liquid nitrogen. The voucher specimens of these materials have been publicly deposited in Shenyang Agriculture University, Shenyang, China. All field experiments were conducted in compliance with the Convention on the Trade in Endangered Species of Wild Fauna and Flora.

### Identification and Phylogenetic Analysis of *ChARF* Family Genes

A local database of hazel protein sequences was constructed using the genomic data of *C. heterophylla*. ARF protein sequences of the model plants *A. thaliana* (AtARF) and *Oryza sativa* (OsARF) were downloaded from the Arabidopsis Information Resource (https://www.arabidopsis.org/) and the Rice Genome Annotation Project (http://rice.plantbiology.msu.edu/), respectively (Wang et al., 2007). The collected sequences were used as queries to search for hazel ARF homologs using BioEdit v7.0.9.0 (Hall, 1999) based on the Basic Local Alignment Search Tool (BLAST)P program (E < 1e-20) in the local hazel database. Meanwhile, the keywords “ARF” and “auxin response factor” were used for direct retrieval on the HazelOmics Database (HOD; http://122.9.151.76/). After eliminating redundant sequences, the Pfam database (https://pfam.xfam.org/search#tabview=tab1) and the Conserved Domain Database (Lu et al., 2020) (https://www.ncbi.nlm.nih.gov/Structure/bwrpsb/bwrpsb.cgi) were used to verify sequences, and the sequences without B3-like DNA-binding (PF02362) and Auxin_resp (PF06507) domains were deleted. ProtParam (Gasteiger et al., 2005) (http://web.expasy.org/protparam/) was used to predict the fundamental physicochemical properties of hazel ARF proteins. WoLF PSORT (https://wolfpsort.hgc.jp) and the Plant-mPLoc Server (Chou and Shen, 2010) (http://www.csbio.sjtu.edu.cn/bioinf/plant-multi/#) were used to predict subcellular localization of hazel ARF proteins.

The grape (Wan et al., 2014) and peach (Li et al., 2016a; Diao et al., 2020) ARF protein sequences (VvARF and PpARF) were collected from the Phytozome v12.0 database (https://phytozome.jgi.doe.gov/pz/portal.html). The Muscle program in the Molecular Evolutionary Genetics Analysis (MEGA, v7.0) software was used to align hazel ARF protein sequences to those from *A. thaliana*, rice, grape, and peach. A neighbor-joining (NJ) tree was constructed based on alignment results using MEGA v7.0 (Kumar et al., 2016), and parameters were set as follows: number of differences model, partial deletion with 95% site coverage cutoff, and bootstrap = 1000. TBtools v1.046 (Chen et al., 2020) was used to display gene structures of hazel *ARF* with the annotation GFF3 file from the HOD. Multiple alignment of hazel ARF proteins was performed using the ClustalW program in the MEGA v7.0 and DNAMAN v9.0.1.116 software. Another phylogenetic tree with only hazel ARF protein sequences was constructed using the maximum-likelihood method with the following parameters: Poisson correction model, partial deletion, and bootstrap = 500. Multiple Em for Motif Elicitation (MEME, v5.2.0) (http://meme-suite.org/tools/meme) (Bailey et al., 2009) was used to predict the conserved domain structure of hazel ARF proteins. The number and the length of motifs were set to 15 and 10–300 amino acids (aa), respectively.

### *ChARF* Expression Profile Analysis

The expression profiles of hazel *ARF* family genes were visualized with a heatmap using EvolView v3 (Subramanian et al., 2019) (https://www.evolgenius.info/evolview/) by compiling the RNA-Seq data with three biological replicates of hazel at four successive ovule developmental stages from the previous study (Liu et al., 2020). The gene expression level was determined according to Fragments Per Kilobase of exon model per Million mapped fragments (FPKM).

### ChARF3 IHC Localization Analysis

More than 18 twigs with only pistillate inflorescences, based on the emasculation and bagging results when the female flower was ready to bloom, were used for IHC. Soon afterward, twigs were transferred to the laboratory, soaked in beakers filled with tap water, and cultured in GXZ-160 incubators with the temperature set to 25°C and light intensity at 900 μmol m^−2^ s^−^1. The flower twigs were divided into three groups with similar inflorescence size, number, and developmental stage. Twenty-four h after artificial pollination using pollen from *C. heterophylla* × *C. avellana* “Bokehong,” female flowers from three groups were infused with 0.1 mg/L indole-3-acetic acid (IAA), 0.5 mg/L 2,3,5-triiodobenzoic acid (TIBA), or distilled water every 24 h, three times, respectively. The three abovementioned solutions contained 0.01% Dow Corning Q2-5211 surfactant. One week after the last treatment, pistillate inflorescences of the pollinated female flowers were sampled, fixed in 4% paraformaldehyde for 16 h at 4°C, and then stored in 70% alcohol at 4°C for subsequent IHC experiments.

Paraffin embedding and slicing of pistillate inflorescences or young fruit followed the method of Liu et al. (2012). Sections were washed in phosphate-buffered saline (PBS) three times for 5 min, microwaved for antigen retrieval, when needed, in 10 mM citric acid buffer (pH 6.0) for 5 min after the liquid started to boil, and then cooled at 25°C. Following pretreatment, sections were blocked with QuickBlock Blocking Buffer (Beyotime, Shanghai, China) for 10 min at 37°C and then incubated overnight at 4°C with the primary polyclonal antibody against ChARF3, diluted in antibody diluent (Beyotime, Jiangsu, China). The specific antibody against a 380 aa long peptide sequence (1–380 aa) containing unique regions in ChARF3 (Supplementary Figure 1) was synthesized by ABclonal Biotechnology Co., Ltd. (Wuhan, China). After three washes in PBS, sections were treated for 30 min at 4°C in PBS containing 0.3% Triton X-100 and 3% NGS and incubated with goat anti-rabbit IgG-horseradish peroxidase (Solarbio, Beijing, China) at a 1:500 dilution. To visualize ChARF3, sections were incubated with diaminobenzidine (0.05 ng/mL, Sigma), 0.01% H_2_O_2_, and 0.15% nickel ammonium sulfate. This reaction was quenched with distilled water when the staining intensity was optimum (5 min). Controls were incubated without the secondary antibody. Finally, the sections were observed and photos were acquired using a light microscope (COIC, Chongqing, China).

### ChARF3 ChIP-Seq Analysis

Fresh ovules from stages Ov1.1, Ov2.1, and Ov3.1 were collected for the ChIP-Seq experiment, which was performed according to previously described methods (Kaufmann et al., 2010; Ricardi et al., 2010). Briefly, the chromatin complexes were isolated and sonicated to shear DNA into 200–600 bp fragments. Thereafter, the specific anti-ChARF3 antibody (ABclonal, Wuhan, China) was used for immunoprecipitation of samples, excluding input controls. After reverse cross-linking, protein digestion, and DNA precipitation, immunoprecipitated DNA was recovered and quantified using a Qubit 4.0 Fluorimeter (*Invitrogen*, Carlsbad, USA). After dilution to 1.5 ng/μL, the DNA quality and insert size were evaluated using agarose gel electrophoresis. Three pairs of input control and antibody-treated ChIP-Seq libraries were constructed and high-throughput sequencing was performed at the Origin-gene Biomedical Technology Co., Ltd. (Shanghai, China), using the Illumina Novaseq 6000 platform. After sequencing six control or antibody-treated libraries, raw reads were subjected to quality filtering using the NGS QC Toolkit (Patel and Jain, 2012) with default parameters. Clean reads were aligned to the reference *C. heterophylla* genome from HOD using Bowtie2 (Langmead et al., 2009) to obtain genome-matched reads. For peak calling, the Bowtie2 alignment output for the six libraries was used together as input for Model-based Analysis of ChIP-Seq (MACS2) (Wu et al., 2013), with a *q*-value threshold of 0.05 to detect peaks (the potential binding sites) of the ChARF3 transcription factor (TF). Subsequently, each peak's signal value in the genome was obtained using the Reads Per Kilobase calculation method per Million mapped reads (RPKM). For each sample, the average signal values of all genes in its library were calculated and the curve and heatmap were created using deepTools (Ramírez et al., 2014).

For *cis*-regulatory element searching, the DNA sequences of the flanking regions from 200 bp upstream and 200 bp downstream of all binding peaks were extracted and analyzed using the Hypergeometric Optimization of Motif EnRichment (HOMER) findMotifsGenome.pl program. The output was then compared to the major databases (HOMER, JASPAR, and other species databases, such as *A. thaliana*) to search for similarities to existing TF-binding motifs. To identify potential ChARF3 target genes, the binding peak sites were associated with the closest protein-coding genes and annotation information of these genes was obtained using the ChIPseeker package (Yu et al., 2015) in R language. Functional enrichment analysis of potential target genes was performed using Gene Ontology (GO) and Kyoto Encyclopedia of Genes and Genomes (KEGG). To gain insight into the target genes regulated by ChARF3, ChIP-Seq and RNA-Seq analyses (Liu et al., 2020) were integrated. DESeq2 was used to identify differentially expressed genes (DEGs) among the three pairwise comparisons of Ov1-vs-Ov2, Ov2-vs-Ov3, and Ov3-vs-Ov4 using a threshold of |log_2_ (fold change)| > 1 and false discovery rate <0.05. Signal plots showing representative peak regions and Venn diagrams were generated using Integrated Genome Viewer v2.8.10 and Calculate and draw custom Venn diagrams (http://bioinformatics.psb.ugent.be/webtools/Venn/), respectively.

## Results

### Identification of the *ChARF* Gene Family

A total of 21 *ARF* candidate genes were selected based on BLASTP search results in the hazel local database and direct retrieval on HOD. Subsequently, 14 non-redundant sequences containing at least B3 and Auxin_resp domains were identified as hazel *ARF* family members (Table 1). The nomenclature system of identified members was set according to their chromosomal location, and they were renamed from *ChARF1* to *ChARF14*. Interestingly, the *ChARF* family members were unevenly distributed on 9 of 11 chromosomes. Three genes were located on chromosome 2; two on chromosomes 3, 4, and 5; and only one on the remaining five chromosomes. The corresponding proteins' length ranged from 639–1,262 aa, and their molecular weight (MW) was predicted to vary from 70.93–139.22 kD. The instability index (II) was larger than 40, suggesting that all the ChARFs were unstable. The average theoretical isoelectric point (pI) and aliphatic index were predicted to be 6.41 and 72.78, respectively. The grand averages of hydropathicity (GRAVY) values were all negative, indicating their hydrophilic character. All 14 proteins were predicted to be located in the nucleus, in accordance with their predicted function as TFs.

**Table 1:** Detailed information of the ARF family genes in hazel.

**Gene**	**Gene ID**	**Location**	**Len (aa)**	**MW (kD)**	**II**	**pI**	**AI**	**GRAVY**	**Domain**	**SCLP**
ChARF1	Cor0171270.1	chr2:7992343 bp−7999406 bp:-	733	79.94	56.17	6.33	70.22	−0.39	B3, ARF	nucl
ChARF2	Cor0138370.1	chr2:18857192 bp−18865334 bp:+	916	101.83	70.26	6.18	74.29	−0.49	B3, ARF, AUX/IAA	nucl
ChARF3	Cor0179220.1	chr2:30101289 bp−30108639 bp:+	690	77.35	56.43	5.86	73.96	−0.35	B3, ARF, AUX/IAA	nucl
ChARF4	Cor0007670.1	chr3:338576 bp−344460 bp:+	748	84.05	58.79	6.29	74.52	−0.50	B3, ARF, AUX/IAA	nucl
ChARF5	Cor0158330.1	chr3:36431850 bp−36443358 bp:–	675	75.36	62.95	5.86	68.41	−0.52	B3, ARF, AUX/IAA	nucl
ChARF6	Cor0126470.1	chr4:299096 bp−304582 bp:+	844	93.96	53.40	6.37	66.30	−0.64	B3, ARF, AUX/IAA	nucl
ChARF7	Cor0082160.1	chr4:26544724 bp−26549732 bp:+	698	78.01	50.38	6.02	72.46	−0.51	B3, ARF, AUX/IAA	nucl
ChARF8	Cor0192010.1	chr5:8936597 bp−8952731 bp:+	851	95.03	58.76	5.93	73.65	−0.47	B3, ARF, AUX/IAA	nucl
ChARF9	Cor0105760.1	chr5:15048490 bp−15089782 bp:–	1,262	139.22	61.27	6.86	79.43	−0.33	B3, ARF, AUX/IAA	nucl
ChARF10	Cor0017310.1	chr6:26567212 bp−26571483 bp:+	639	70.93	49.89	8.10	74.27	−0.41	B3, ARF	nucl
ChARF11	Cor0181440.1	chr8:2580200 bp−2587490 bp:+	935	103.23	55.79	5.27	76.49	−0.39	B3, ARF, AUX/IAA	nucl
ChARF12	Cor0045580.1	chr9:14085665 bp−14088920 bp:+	711	78.28	47.62	8.16	73.49	−0.36	B3, ARF	nucl
ChARF13	Cor0131740.1	chr10:22066080 bp−22075437 bp:–	1,137	127.06	70.88	6.12	72.15	−0.69	B3, ARF, AUX/IAA	nucl
ChARF14	Cor0002660.1	chr11:16147771 bp−16152282 bp:+	703	77.25	50.50	6.40	69.23	−0.37	B3, ARF	nucl

*Len, length, aa, number of amino acids; MW, molecular weight; pI, theoretical isoelectric point; II, Instability index; AI, aliphatic index; GRAVY, grand average of hydropathicity; B3, B3-like DNA-binding domain (PF02362), ARF, Auxin_resp domain (PF06507), AUX/IAA, Aux/IAA domain (PF02309); SCLP, Subcellular localization prediction; nucl, nucleus*.

### Phylogenetic and Structural Analyses of *ChARFs*

**Figure 1:**
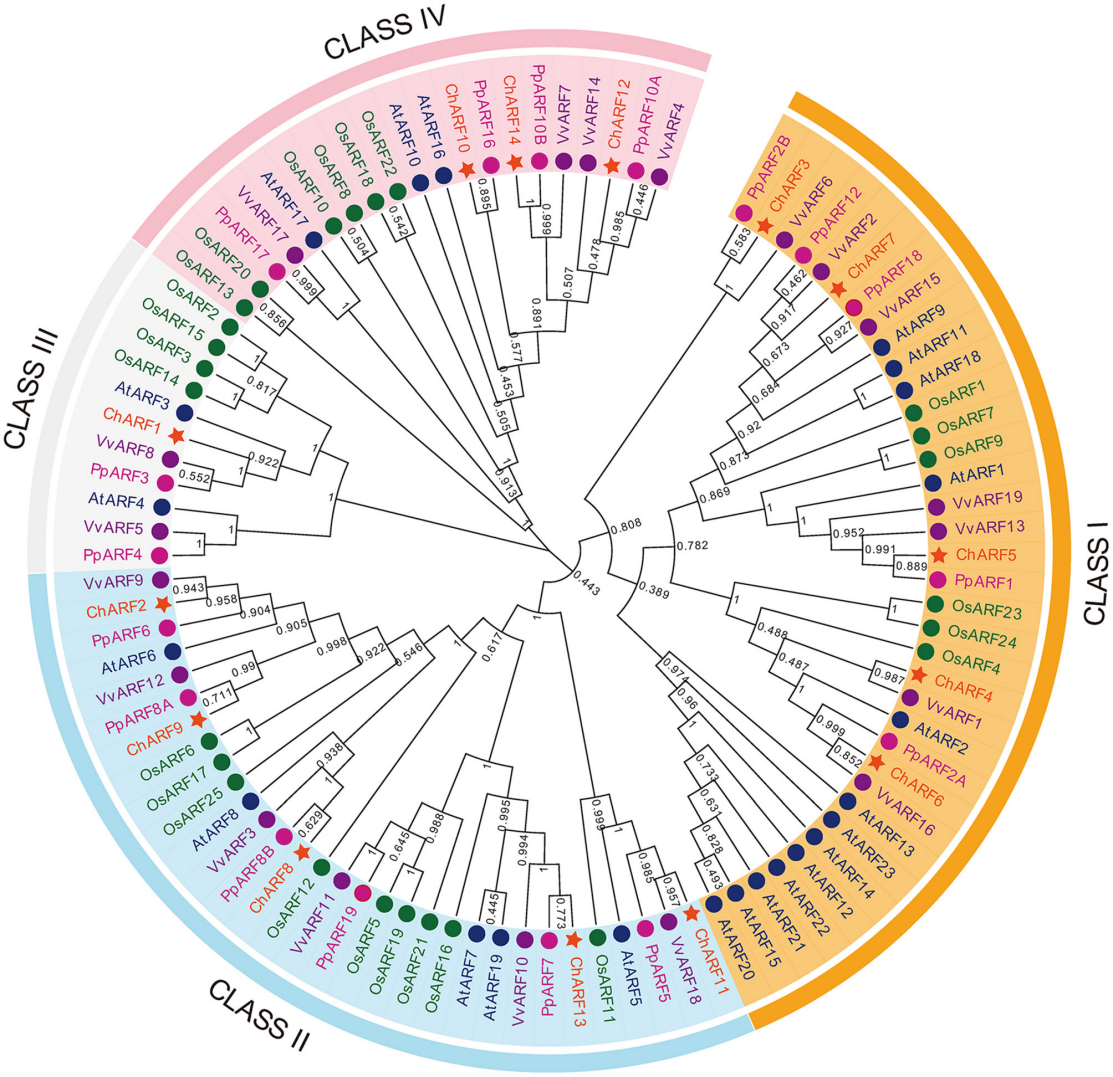
Phylogenetic analysis of *Arabidopsis*, grape, peach, rice, and hazel ARF proteins. Amino acid sequences of full-length predicted ARF proteins were aligned using MUSCLE program. Phylogenetic tree was generated using MEGA v7.0 program via neighbor-joining method with 1,000 Bootstrap values.

To further investigate the phylogenetic relationships between the ChARF family members, an NJ tree was constructed by aligning the 14 ChARF sequences with 23 AtARF, 25 OsARF, 17 PpARF, and 19 VvARF sequences from *A. thaliana*, rice, peach, and grape, respectively. Ninety-eight ARFs were divided into four groups comprising Class I to IV (Figure 1). Among all ChARFs, five members (ChARF3/4/5/6/7) belonged to Class I and had a relatively close genetic relationship with repressors AtARF1/2/9/11/18. Notably, ChARF3 was in an isolated branch with PpARF2B and VvARF6 without any AtARFs or OsARFs, suggesting obvious differences between *A. thaliana* and hazel *ARF* genes. Class II members included all five activators, ChARF2/8/9/11/13, homologous to AtARF5/6/7/8/19. Only one member (ChARF1) in Class III shared high sequence similarity with AtARF3, PpARF3, and VvARF8. The remaining three members (ChARF10/12/14) were included in Class IV. Generally, the phylogenetic distribution results indicated higher homology between hazel and peach.

**Figure 2:**
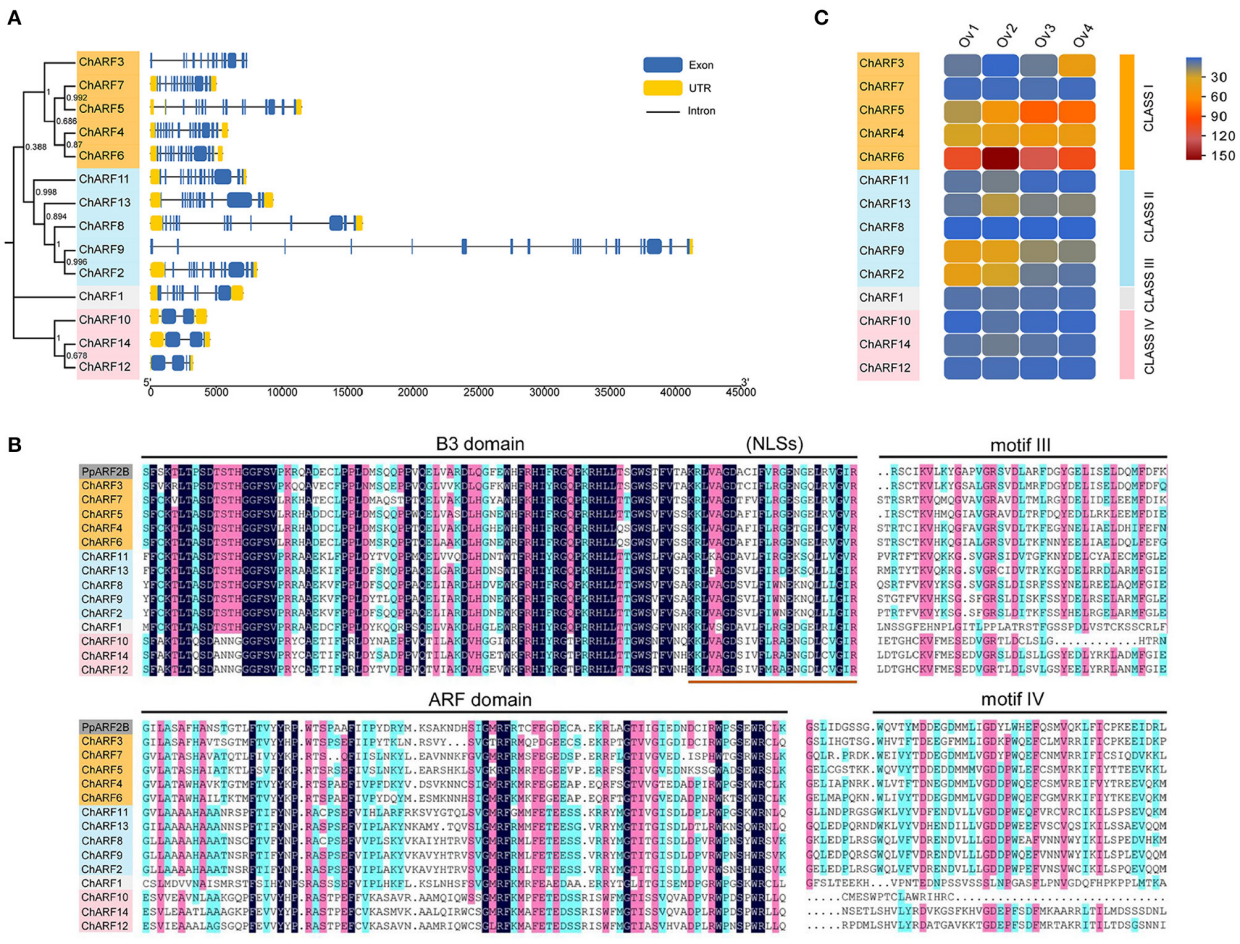
Gene structures, multiple sequence alignment and expression pattern analysis. **(A)** Gene structures of *ChARF* family genes. The untranslated region (UTR), exon and intron are represented by yellow box, blue box and black line, respectively. **(B)** Multiple sequence alignment of *ChARF* family proteins. The B3 domain, Auxin_resp (ARF) domain, motif III and motif IV domain are marked by lines. The ends of the B3 domains include classical nuclear localization signals (NLSs). **(C)** Expression pattern heatmap of *ChARF* family genes during ovule developmental stages. Expression is normalized based on the value of fragments per kilo base of exon per million fragments mapped (FPKM) and the heatmap was constructed by Evolview online software.

The structure of each *ChARF* was displayed using Tbtools software (Chen et al., 2020), according to the annotation GFF3 file from the HOD (Figure 2A). The number of introns in *ChARF* genes ranged from 1–19. The *ChARF* genes in Classes I, II, and III harbored 9–19 introns, whereas the *ChARF* genes in Class IV only contained 1–3 introns. The length of the former eight introns within *ChARF8* was more than 30 kb, which was similar to *VvARF3*. ClustalW, DNAMAN, and MEME were used to perform a multiple alignment to identify the conserved motifs of the ChARF protein sequences (Figure 2B; Supplementary Figure 2). The B3 domain, harboring motif 1, was highly conserved in the *ChARF* gene family and contained the nuclear location signal at the C-terminal. Aux/IAA domain (PF02309) containing motifs III and IV and Auxin_resp domain were less conserved. Motifs 6/7/3 and motifs 13/8/5 belonged to the Auxin_resp and Aux/IAA domain, respectively. All ChARFs, except ChARF1/10, contained a C-terminal Aux/IAA domain, whereas ChARF12/14 harbored a partial Aux/IAA domain. Taken together, the ChARF members harboring similar conserved motifs tended to be distributed in the same classes.

### *ChARF* Expression Levels in the Ovule

To explore the potential functions of *ChARF*s in the ovule, FPKM values were used to determine the expression profiles of 14 *ChARF* genes at stages Ov1 to Ov4 (Figure 2C). During four ovule developmental stages, the expression abundance of seven *ChARFs* (*ChARF1/7/8/10/12/14*) remained at a low level (average expression level <10), and no significant differences in expression were detected between adjacent stages, suggesting that these *ChARFs* might not have major roles in ovule development. The remaining *ChARFs* (*ChARF2/3/4/5/6/9/11/13*) showed relatively higher expression at one or several developmental stages, indicating that they were associated with ovule development. These seven *ChARFs*, transcriptional repressors (*ChARF3/4/5/6*) and activators (*ChARF2/9/13*), were distributed in Class I and Class II, respectively. Among them, *ChARF4* was constitutively expressed in all four stages, and *ChARF2/9* were expressed in the first two stages, while *ChARF3/5/6/13* displayed significant expression differences between adjacent stages and continuous expression changes from stages Ov1 to Ov4, inferring that they might mediate ovule development. *ChARF3*, a repressor, was downregulated 3.67-fold from stage Ov1 to stage Ov2 and upregulated 6.44-fold from stage Ov2 to stage Ov4, showing the lowest expression level at stage Ov2 of early ovule growth (containing ovule fertilization process) and the most significant differences among the four stages. This finding suggested that *ChARF3* might coordinate ovule development, especially ovule fertilization within stage Ov2, by reducing the inhibition of its downstream genes.

### ChARF3 Spatiotemporal Expression by IHC Localization

**Figure 3:**
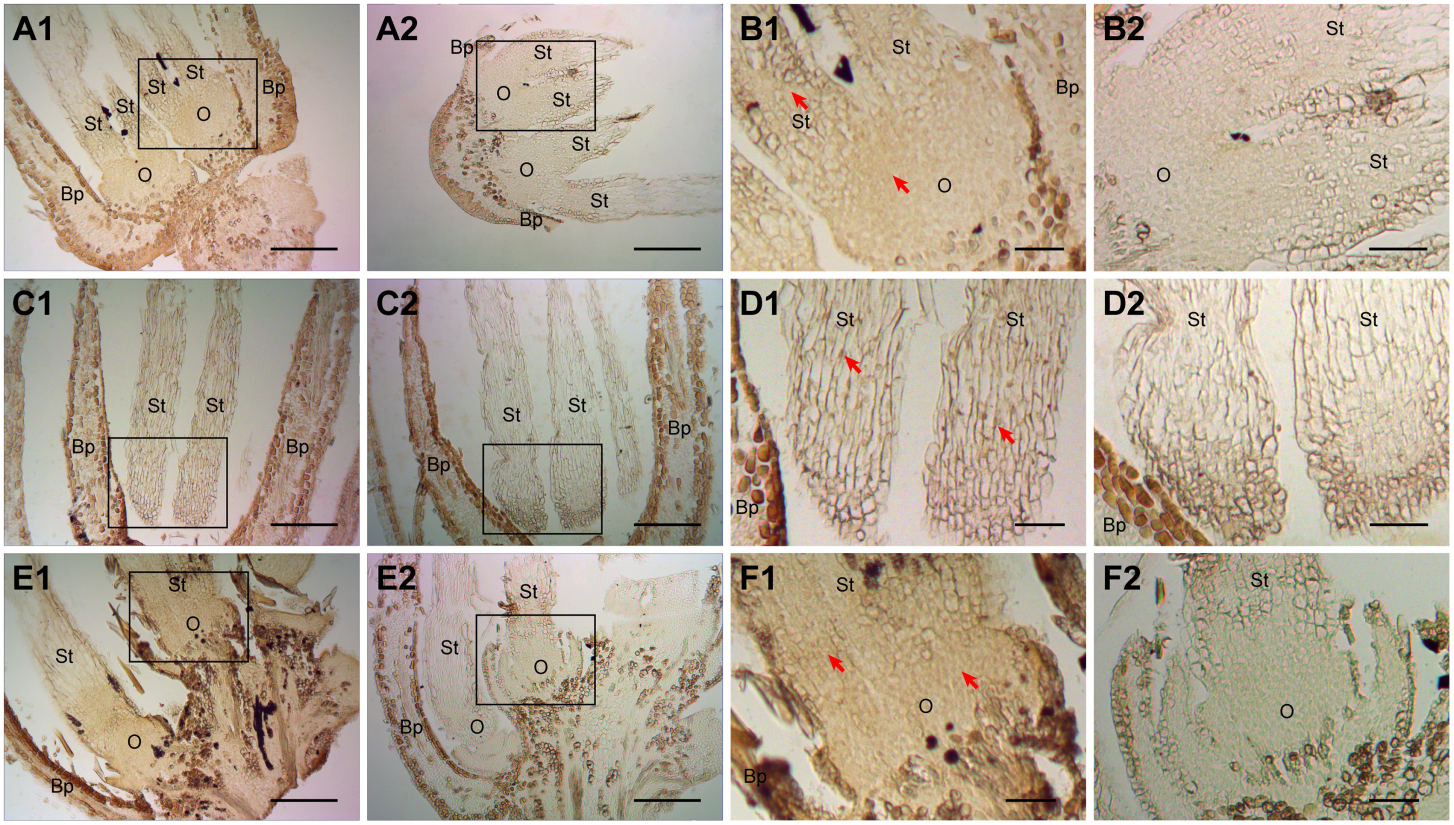
Effects of IAA and TIBA on ChARF3 localization after pollination. **(A1, C1, and E1)** Female flowers treated with IAA, TIBA, or distilled water, respectively. **(B1, D1, and F1)** The enlarged views of black squares in A1, C1, and E1, respectively. **(A2–F2)** The control of A1 to F1 without secondary antibody treatment, respectively. Key: Bp, bract primordium; O, ovary; St, style. Scale bars: A, C, E = 200 μm; B, D, F = 50 μm. Positive staining is shown in brown. Red arrows indicate the localized areas.

To further verify whether ChARF3 is induced by auxin and involved in ovary initiation regulation, the anti-ChARF3 polyclonal antibody was prepared, followed by IHC localization (Figure 3). Female flowers after artificial pollination were treated with 0.1 mg/L IAA, 0.5 mg/L TIBA, or distilled water. In female flowers treated with IAA (Figures 3A1,A2,B1,B2) and distilled water (Figures 3E1,E2,F1,F2), the early ovary primordium cell number was much more than that in the TIBA treatment (Figures 3C1,C2,D1,D2), and more ChARF3 accumulated in ovary primordium cells than in those of the TIBA treatment. Though the exogenous application of IAA did not promote the expression of ChARF3 in ovary primordium cells (Figures 3A1,B1,E1,F1), TIBA, which works as an IAA transport inhibitor, inhibited cell proliferation in the ovary primordium and ChARF3 expression. Collectively, these results suggested that the development of ovary primordium and ChARF3 expression were both regulated by IAA in hazel.

**Figure 4:**
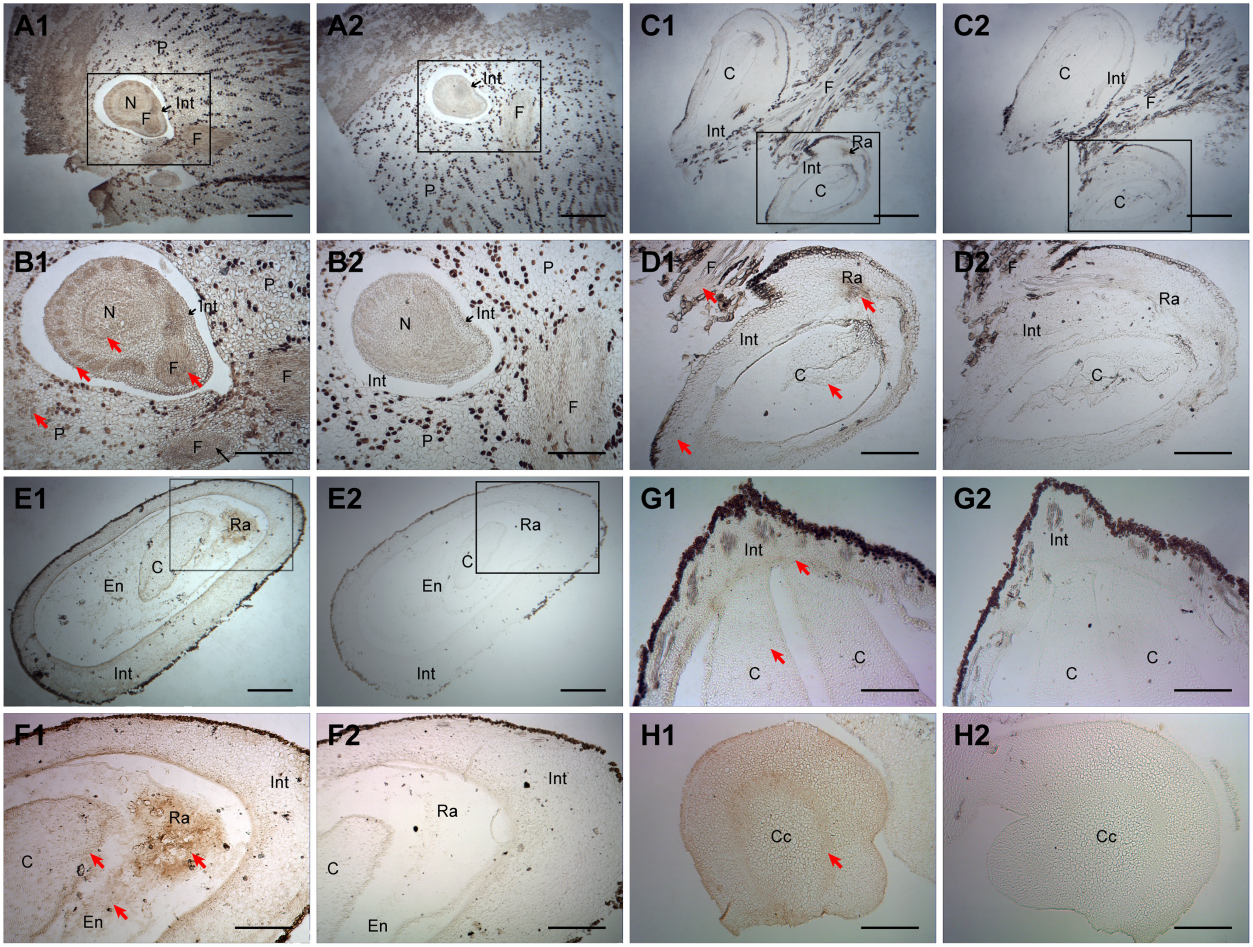
Immunohistochemical analysis of ChARF3 localization during ovule development. **(A1, A2, B1, and B2)** Before fertilization at stage Ov1.1, the ChARF3 staining of funiculus, integument, and parenchyma is deeper, 40 days after blooming. B1 and B2 are enlarged views of black squares in A1 and A2, respectively. **(C1, C2, D1, and D2)** After fertilization at stage Ov2.1, the ChARF3 staining of funiculus, integument, radicle, and cotyledon is obvious, 70 days after blooming. D1 and D2 are enlarged views of black squares in C1 and C2, respectively. **(E1, E2, F1, and F2)** During growth of the ovule at stage Ov3.1, the ChARF3 staining is enriched in the cotyledon, endosperm, and radicle, 100 days after blooming. F1 and F2 are enlarged views of black squares in E1 and E2, respectively. **(G1, G2, H1, and H2)** The ChARF3 staining keep higher in the cotyledon and radicle, 100 days after blooming. **(A2–H2)** The control of A1 to H1 without secondary antibody treatment, respectively. Key: Cc: central cylinder; C: cotyledon; En: endosperm; F, funiculus; Int, integument; N, nucellus; P, parenchyma; Ra: radicle; St, style. Scale bars: A, C, E = 400 μm; B, D, F, G, H = 200 μm. Positive staining is shown in brown. Red arrows indicate the localized areas.

Subsequently, ChARF3 IHC localization in ovary or ovule samples was examined by microscopy (Figure 4). At the ovule formation stage Ov1.1 (before fertilization), ChARF3 was especially expressed in the funiculus, integument, and parenchyma (Figures 4A1,A2,B1,B2). At stage Ov2.1 (early ovule growth), hazel fertilization was complete, evidenced by the obvious cotyledon embryo in the ovule (Figures 4C1,C2,D1,D2). ChARF3 was widely expressed in the funiculus, integument, radicle, and cotyledon (Figures 4C1,C2,D1,D2). During stage Ov3.1 (rapid ovule growth), ChARF3 was enriched in the cotyledon, endosperm, and radicle, and its expression in the radicle was most abundant (Figures 4E1,E2,F1,F2). Subsequently, ChARF3 expression in the cotyledon remained higher than that in the control (Figures 4G1,G2). In the radicle transverse section, ring-shaped staining was observed around the central cylinder (Figures 4H1,H2). These results demonstrated that ChARF3 was expressed in the ovule, consistent with the high expression at the transcriptional level observed in the ovule (Figure 2C), suggesting its potential role in regulating ovule development.

### Genome-Wide Detection of ChARF3-Binding Sites via ChIP

To identify ChARF3-binding sites, a ChIP experiment was performed using the specific polyclonal antibody against ChARF3. Three pairs of the input control and antibody-treated ChIP-Seq libraries were constructed using ovules from three developmental stages, Ov1.1, Ov2.1, and Ov3.1 (Table 2), and high throughput sequencing was performed. For the six libraries from the three stages, a total of ~32–62 million high-quality clean reads were obtained, of which ~51.00%−83.96% were mapped to the hazel genome using Bowtie 2 (Table 2). On average, the percentages of unique and multi-mapping reads were 36.24 and 35.21, respectively. The proportion of reads mapping to positive chains was equivalent to the proportion of reads mapping to negative chains in the hazel genome. These results suggested that the genome-wide mapping of binding sites was reliable.

**Table 2:** Summary of ChIP-Seq reads from six libraries matched to the hazel (*Corylus heterophylla* Fisch.) genome.

**Sample**	**Clean reads**	**Total map**	**Unique map**	**Multi map**	**Positive map**	**Negative map**
Ov1.1-ChIP	38,097,464	25,585,897 (67.159%)	13,892,761 (36.466%)	11,693,136 (30.693%)	6,945,144 (18.230%)	6,947,617 (18.236%)
Ov1.1-Input	62,043,222	42,156,639 (67.947%)	16,840,975 (27.144%)	25,315,664 (40.803%)	8,413,937 (13.561%)	8,427,038 (13.583%)
Ov2.1-ChIP	38,079,134	30,312,827 (79.605%)	16,282,502 (42.760%)	14,030,325 (36.845%)	8,135,004 (21.363%)	8,147,498 (21.396%)
Ov2.1-Input	50,194,378	42,143,941 (83.961%)	23,487,361 (46.793%)	18,656,580 (37.169%)	11,742,531 (23.394%)	11,744,830 (23.399%)
Ov3.1-ChIP	37,139,614	18,940,594 (50.998%)	9,736,568 (26.216%)	9,204,026 (24.782%)	4,862,846 (13.093%)	4,873,722 (13.123%)
Ov3.1-Input	32,329,742	25,553,921 (79.042%)	12,301,206 (38.049%)	13,252,715 (40.992%)	6,147,990 (19.017%)	6,153,216 (19.033%)

*Ov1.1, ovule formation, Ov2.1, early ovule growth, Ov3.1, rapid ovule growth; ChIP, chromatin-immunoprecipitated DNA samples; Input: DNA samples before shearing*.

**Figure 5:**
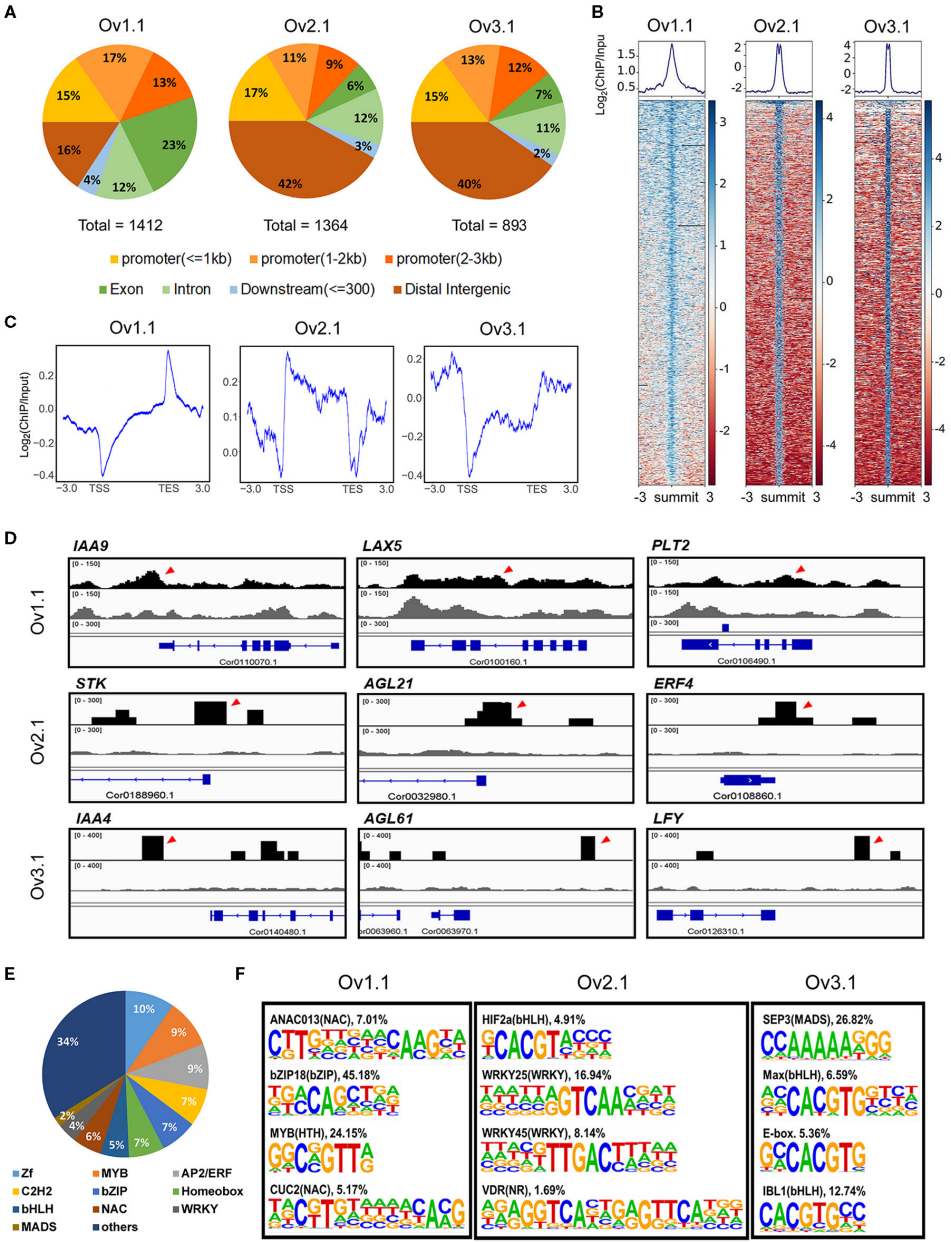
Genome-wide analysis of ChARF3-binding sites identified by ChIP-Seq at three ovule development stages. **(A)** Distribution of ChARF3-binding sites in different genomic locations (exon, intron, promoter, downstream, and intergenic regions). **(B)** Heatmaps of ChIP-seq signal densities of ChARF3 showing enrichment near the summit of peaks. The value of Log_2_(ChIP/Input) reflects the intensity of the binding sites in ChIP samples compared with Input controls. **(C)** Distribution of ChARF3-binding sites near transcription start site (TSS) and transcription end site (TES) regions. **(D)** Integrative Genomics Viewer (IGV) visualization of representative examples of ChIP-seq binding profiles at selected targeted sites. **(E)** Distribution of transcription factors across all *cis*-regulatory motifs. **(F)** Significantly enriched *cis*-regulatory motifs among the ChARF3-binding sites at three ovule development stages. Log_2_*P*-value < −4.32.

To call peaks corresponding to the ChARF3-binding sites, the mapping outputs for both input control and antibody-treated libraries were used together as input into the MACS software. A total of 1,412, 1,364, and 893 peaks representing potential ChARF3-binding sites were detected at stages Ov1.1, Ov2.1, and Ov3.1, respectively (Supplementary Table 1; Figure 5A), showing a declining trend with ovule development. At stage Ov1.1, ChARF3 preferentially bound to exons and promoters (≤1 kb), and the percentage of ChARF3 binding to intergenic regions and promoters (≤1 kb) was 31.09%, which was much lower than the average percentage of 56.90% at stages Ov2.1 and Ov3.1 (Figure 5A). Therefore, the ChARF3-binding region changed significantly during different developmental periods. ChARF3-binding signal densities valued by RPKM in the clustering heatmap were highly enriched in the peak summits across the treated samples compared to the input controls (Figure 5B), suggesting that the results of the ChIP analysis were reliable. Meanwhile, ChARF3-binding sites resided in transcription start site regions (-3 kb to 3 kb) and transcription end site regions (−3 to 3 kb) at three developmental stages (Figure 5C), indicating that ChARF3 bound to *cis*-regulatory elements of target genes and regulated their expression at the transcriptional level. Key ChARF3 target genes, which might be involved in the regulation of flower and fruit development, were chosen and their position in the hazel genome was displayed using the Integrative Genomics Viewer (Figure 5D), including *AUXIN/INDOLE ACETIC ACID 4/9* (*IAA4/9*, Cor0140470.1 and Cor0110070.1), *AUXIN INFLUX CARRIER PROTEIN* (*AUX1*)-*LIKE PROTEIN 5* (*LAX5*, Cor0100160.1), *AGAMOUS LIKE 21/61* (*AGL21/61*, Cor0032980.1 and Cor0063970.1), *AP2-like ethylene-responsive transcription factor* (*AP2/ERF*) *PLT2* (*PLT2*, Cor0106490.1), *Ethylene Responsive Factor 4* (*ERF4*, Cor0108860.1), *LEAFY* (*LFY*, Cor0126310.1), and *SEEDSTICK/AGAMOUS LIKE 11* (STK/AGL11, Cor0188960.1).

In total, 823 *cis*-regulatory elements were identified from the ChARF3 binding peaks of three ovule developmental stages (Supplementary Table 2), and the top abundant DNA motifs located in the genes encoding zinc finger, MYB, AP2/ERF, Cys2His2-like, and basic leucine zipper (bZIP) TFs, which accounted for 10%, 9%, 9%, 7%, and 7% of all motifs, respectively (Figure 5E; Supplementary Table 2). The motifs in genes encoding MADS-box, AP2/ERF, and ARF TFs, which are expected to regulate flower and fruit development, accounted for approximately 11% of all identified motifs (Figure 5E; Supplementary Table 2). Among these, the *ChARF3* motif [TTGTCGG(A/C)(A/T)(A/T/G/C)] accounted for 17.32%−20.18% of target sequences in the three developmental stages, showing a stage specificity pattern (Supplementary Table 2). At specific stages during ovule development, ChARF3 tended to bind genes harboring different *cis*-regulatory motifs. For example, the significantly enriched *CUP-SHAPED COTYLEDON2* [T(A/G)C(G/T)TGT(A/T/G/C)(A/T/G/C)(A/T/G/C)(A/T) CA(A/C)G], *WRKY25* [(A/T/G/C)(A/T/C)(A/G)GTCAA (A/C)(A/T/G/C)], and *SEPALLATA3* [CCAAAAAGGG] motifs identified in Ov1.1, Ov2.1, and Ov3.1, respectively (Figure 5F; Supplementary Table 2), suggested that ChARF3 might target different genes to coordinate ovule development at different stages.

### Identification of ChARF3 Target Genes

**Figure 6:**
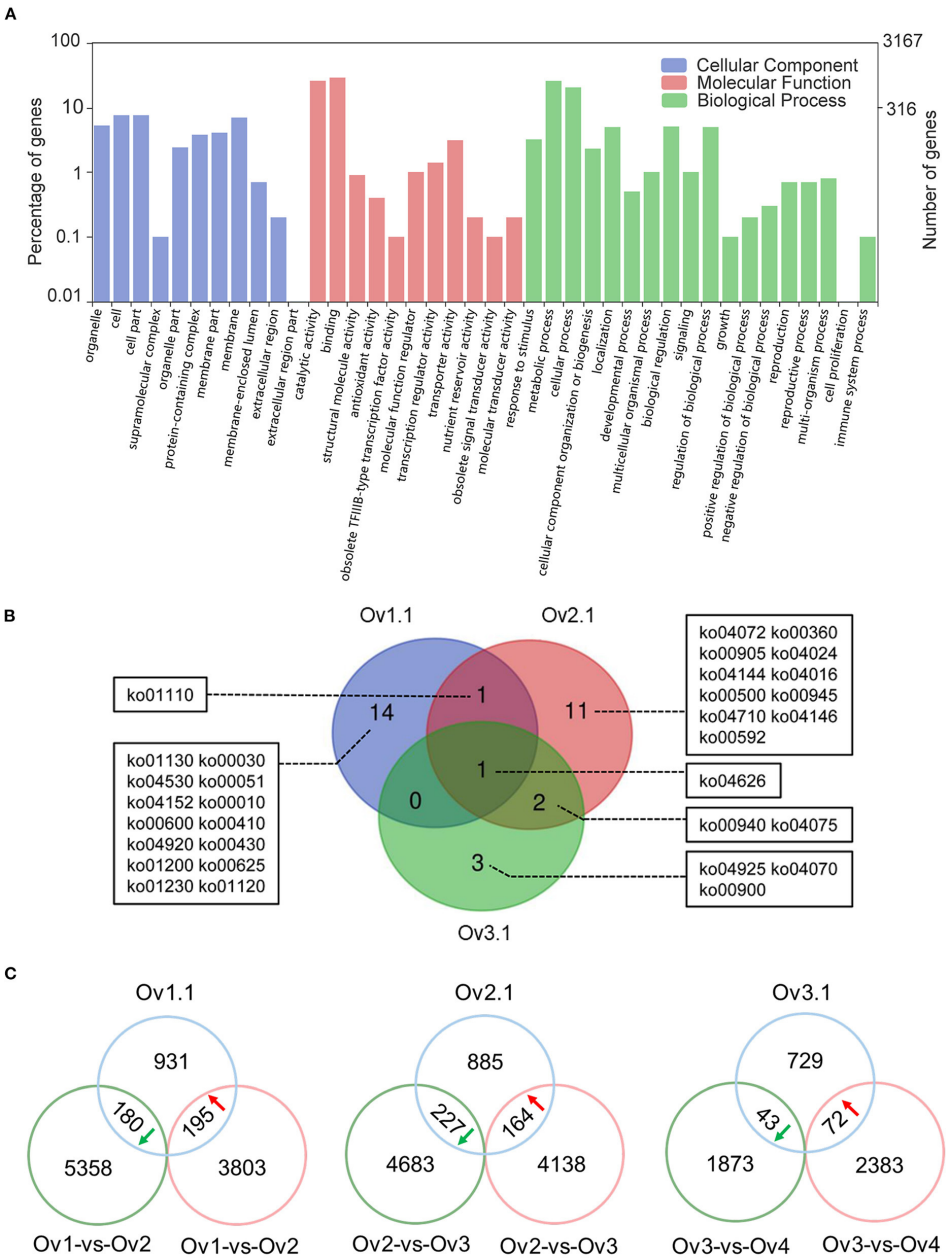
GO and KEGG enrichment and ChARF3-regulated genes analysis. **(A)** GO classification of all ChARF3 target genes. **(B)** Overlaps of significantly enriched KEGG pathways at three ovule development stages. ko00010: Glycolysis/Gluconeogenesis, ko00030: Pentose phosphate pathway, ko00051: Fructose and mannose metabolism, ko00360: Phenylalanine metabolism, ko00410: beta-Alanine metabolism, ko00430: Taurine and hypotaurine metabolism, ko00500: Starch and sucrose metabolism, ko00592: alpha-Linolenic acid metabolism, ko00600: Sphingolipid metabolism, ko00625: Chloroalkane and chloroalkene degradation, ko00900: Terpenoid backbone biosynthesis, ko00905: Brassinosteroid biosynthesis, ko00940: Phenylpropanoid biosynthesis, ko00945: Stilbenoid, diarylheptanoid and gingerol biosynthesis, ko01110: Biosynthesis of secondary metabolites, ko01120: Microbial metabolism in diverse environments, ko01130: Biosynthesis of antibiotics, ko01200: Carbon metabolism, ko01230: Biosynthesis of amino acids, ko04016: MAPK signaling pathway—plant, ko04024: cAMP signaling pathway, ko04070: Phosphatidylinositol signaling system, ko04072: Phospholipase D signaling pathway, ko04075: Plant hormone signal transduction, ko04144: Endocytosis, ko04146: Peroxisome, ko04152: AMPK signaling pathway, ko04530: Tight junction, ko04626: Plant-pathogen interaction, ko04710: Circadian rhythm, ko04920: Adipocytokine signaling pathway, ko04925: Aldosterone synthesis and secretion. **(C)** Overlap between ChIP-Seq target genes and RNA-Seq DEGs. Green and red circles refer to down- and up-regulated differentially expressed genes in paired comparison respectively, and blue circle refers to genes bound by ChARF3 through ChIP-seq.

The genes covered by or adjacent to overlapping peaks might be target genes regulated by ChARF3. The binding peak regions were associated with the closest protein-coding genes using the ChIPseeker package, and 1,306, 1,276, and 844 potential ChARF3 target genes at stages Ov1.1, Ov2.1, and Ov3.1 were identified (Supplementary Table 1). In total, 3,167 non-redundant ChARF3 target genes were analyzed using GO annotation, among which 1,780 genes (56.20%) were mapped to GO terms (Figure 6A). Most annotated target genes were located in the cell membrane and organelles, enriched in molecular function of binding and catalytic activity, and associated with biological processes related to metabolic process, cellular process, and biological regulation. KEGG pathway enrichment analysis revealed that ChARF3 target genes were significantly enriched in 16, 15, and 6 pathways at stages Ov1.1, Ov2.1, and Ov3.1, respectively (Supplementary Table 3). A Venn diagram was constructed to illustrate the unique and common pathways (Figure 6B). All three stages shared the common significantly enriched ko04626 pathway (plant–pathogen interaction). The ko01110 pathway (biosynthesis of secondary metabolites) was common in stages Ov1.1 and Ov2.1. The ko00940 (phenylpropanoid biosynthesis) and ko04075 (plant hormone signal transduction) pathways were common in stages Ov2.1 and Ov3.1 (Table 3). GO and KEGG enrichment analysis results for the ChARF3 target genes suggested that ChARF3 might regulate the expression of target genes in pathways including biosynthesis of metabolites, disease resistance, hormone signal transduction, and coordinated ovule development in hazel.

**Table 3:** Overlapping significant enriched KEGG pathways at three ovule development stages in hazel.

**Term ID**	**Category**	**Gene Count**			**Enrich factor**			***P*** **-value**		
		**Ov1.1**	**Ov2.1**	**Ov3.1**	**Ov1.1**	**Ov2.1**	**Ov3.1**	**Ov1.1**	**Ov2.1**	**Ov3.1**
ko04626	Organismal Systems	8	8	5	2.16	2.61	2.56	2.42E-02	8.07E-03	4.13E-02
ko01110	Metabolism	65	56		1.29	1.34		1.13E-02	7.95E-03	
ko00940	Metabolism		8	6		3.85	4.52		4.71E-04	1.33E-03
ko04075	Environmental Information Processing		12	7		2.68	2.44		9.65E-04	2.11E-02

*Ov1.1, ovule formation, Ov2.1, early ovule growth, Ov3.1, rapid ovule growth. ko04626, Plant-pathogen interaction; ko01110, Biosynthesis of secondary metabolites; ko00940, Phenylpropanoid biosynthesis; ko04075, Plant hormone signal transduction*.

ChARF3 acts as a transcriptional repressor in the auxin signal transduction pathway, and its binding inhibits the expression of downstream auxin-induced genes. If *ChARF3* expression is upregulated, its target gene expression will be downregulated and vice versa, and the trend in *ChARF3* expression changes is expected to be opposite to that of its target genes. Combination analysis of ChARF3 ChIP-Seq and RNA-Seq data from different ovule developmental stages (Liu et al., 2020) revealed that 881 target genes from stages Ov1.1, Ov2.1, and Ov3.1 were differentially expressed (Figure 6C; Supplementary Table 4). Among these, 195 upregulated, 227 downregulated, and 43 downregulated genes showing opposite trends in expression changes with *ChARF3* in the Ov1-vs-Ov2, Ov2-vs-Ov3, and Ov3-vs-Ov4 paired comparisons were identified, suggesting they were potential target genes regulated by ChARF3. Based on these data, a set of 49 candidate ChARF3 target genes were identified that were involved in auxin synthesis, transport, signaling pathways, and ovule development (Supplementary Table 5).

## Discussion

As a principal phytohormone of growth and development, auxin controls a variety of diverse responses in plants (Woodward and Bartel, 2005). ARFs are essential proteins in auxin-mediated pathways and have critical roles in plant growth, development, and stress responses, including reproductive organ development. In this study, the recently developed reference genome database on the HOD was employed to identify and analyze the members of the hazel *ARF* gene family, using a combination of bioinformatics and experimental approaches, revealing the physicochemical properties (Table 1), phylogenetic relationship (Figure 1), and structural characterization (Figures 2A,B) of the *ChARF* gene family. Based on previous RNA-Seq data (Liu et al., 2020), 14 *ChARFs* were identified in hazel. Among these, *ChARF3*, a repressor, showed the lowest expression level at stage Ov2 of early ovule growth (containing ovule fertilization process) and the most significant differences among the four stages, suggesting that *ChARF3* might coordinate ovule development by reducing the inhibition of its downstream genes. *ChARF3* was homologous to *PpARF2B* and *VvARF6* and was classified into Class I *AtARF1/2* (Figure 1). Additionally, IHC localization was performed, suggesting that auxin was essential for ovary initiation and that ChARF3 was enriched in the vigorous growth area of the ovary and ovule, including the ovary primordium, funiculus, integument, radicle, and cotyledon (Figures 3, 4). Combining the gene family, gene expression, and IHC analysis results, it was hypothesized that ChARF3 regulated the ovary and ovule development processes. Subsequently, genome-wide binding sites and genes mediated by ChARF3 were identified via ChIP-Seq and RNA-Seq, and target genes with a trend in expression changes that were opposite to that of *ChARF3* were examined. These findings provided a better understanding of hazel ovule development regulation.

### ChARF3 Targeted Auxin Biosynthesis, Transport, and Signal Transduction Genes

Indole-3-pyruvate monooxygenase YUCCA10 (YUC10), LAX5, and the efflux carrier protein likes 7 (PIN7) have key roles in auxin biosynthesis, transport, and distribution (Schrader et al., 2003). In maize, YUC10 mediates auxin biosynthesis in the embryo sac (Chettoor and Evans, 2015). In *Arabidopsis*, early axis formation requires PIN7-mediated auxin asymmetry during embryogenesis (Xiong et al., 2019). *YUC10* (Cor0034390.1 and Cor0179200.1) was 8.96-fold upregulated and 1.19-fold downregulated in the Ov1-vs-Ov2 and Ov2-vs-Ov3 comparisons, respectively. *LAX5* (Cor0100160.1) and *PIN7* (Cor0204530.1), which were ChARF3 target genes in stages Ov1.1 and Ov2.1, were upregulated and downregulated in the Ov1-vs-Ov2 and Ov2-vs-Ov3 comparisons, respectively. *YUC10, LAX5*, and *PIN7* expression changes might change auxin distribution in the ovule, and ChARF3 was hypothesized to be involved in the regulation of auxin biosynthesis and transport through its regulatory effect on these genes.

ARFs are responsible for the regulation of expression of early auxin response genes, including *Aux/INDOLE-3-ACETIC ACID* (*AUX/IAAs*) and *SMALL AUXIN-UP RNAs* (*SAURs*) (Hagen and Guilfoyle, 2002). In *Arabidopsis* and tomato, IAA4/9/30 are pivotal mediators of auxin in fruit initiation and embryo maturation (Wang et al., 2005; Braybrook et al., 2006; Goetz et al., 2006; Pomares-Viciana et al., 2019). Among ChARF3 targets, *IAA9* (Cor0110070.1) and *IAA30* (Cor0096370.1) were 1.24- and 2.43-fold upregulated in the Ov1-vs-Ov2 comparison, and *IAA4s* (Cor0086990.1 and Cor0140470.1) was 4.05- and 1.40-fold downregulated in the Ov2-vs-Ov3 and Ov3-vs-Ov4 comparisons, respectively. Cell expansion is a fundamental process essential for plant growth and development, and SAURs modulate polar auxin transport and play a key role in cell expansion (Weijers et al., 2006). In *Arabidopsis*, SAUR32 negatively regulates cell expansion (Park et al., 2007). At stages Ov1 and Ov2, the *SAUR32* (Cor0157210.1 and Cor0036810.1) expression was relatively higher. At stage Ov2.1, *SAUR32* (Cor0036810.1) was targeted by ChARF3, and its expression was inhibited by 2.15-fold in the Ov2-vs-Ov3 comparison, which might be beneficial for cell expansion in the ovule, an essential process underlying fruit initiation. These results indicated that ChARF3 mediated fruit initiation by targeting *IAA4/9/30* and *SAUR32*.

### ChARF3 Targeted Important Regulators of Ovule Development

Several classes of MIKCc-type genes, called floral MADS-box genes, are involved in the regulation of floral component (organs) development and flowering time (Weigel and Meyerowitz, 1994; Theissen et al., 2000). According to the floral quartet model, floral organ identity of the carpel and ovule is determined by specific combinational quaternary complexes consisting of AG-AG-SEP-SEP and SEP-AG-STK-SHP heterodimers, respectively (Theissen, 2001; Maejima et al., 2014). In rice, *OsMADS6/MOSAIC FLORAL ORGANS 1* (*MFO1*) belongs to the *AGL6* clade, a sister clade to E function SEP-like genes (Li et al., 2010). *AGL*6-like genes regulate carpel and ovule development and floral meristem determinacy (Li et al., 2011). Cor0152110.1, a gene homologous to *OsMADS6/MFO1*, was downregulated 1.57-fold in Ov2-vs-Ov3. In *Arabidopsis*, Seedstick/Agamous like 11 (STK/AGL11) is a key TF that controls ovule identity, with its RNA accumulating in developing ovules (Rounsley et al., 1995; Pinyopich et al., 2003). Similarly, STK of the woody plant *Prunus persica* is also important for embryo development (Tani et al., 2009). In hazel, STK encoded by Cor0188960.1 showed the highest expression at stage Ov2, ChARF3 bound to its promoter (<=1 kb) at Ov2.1, and gene expression decreased by 2.13-fold. *AGAMOUS* subfamily members associate with reproductive organ identity determination, fruit, seed development, and cell specification (Gong et al., 2004; Yu et al., 2014). At stage Ov2.1 and Ov3.1, *AGL21* (Cor0032980.1) and *AGL61* (Cor0063970.1) expression decreased by 2.51- and 1.43-fold in Ov2-vs-Ov3 and Ov3-vs-Ov4 pairwise comparisons, respectively. Collectively, ChARF3 regulated ovule development by coordinating the expression of *MADS* genes, including *MFO1, STK, AGL21*, and *AGL61*.

Many important TFs, such as AP2-like ethylene-responsive transcription factor (AP2/ERF) PLT2 (PLT2), LEAFY (LFY), FT-interacting protein (FT), suppressor of constans overexpression 1 (SOC1), and transcription factor TCP (TCP), participate in flower and fruit development involving cell division and proliferation (Nagpal et al., 2005; Poza-Carrión et al., 2007; Mähönen et al., 2014) as well as flowering decision and time (Lee and Lee, 2010). At stages Ov1.1 to Ov3.1, ChARF3 bound to *PLT2* (Cor0106490.1), *LFY* (Cor0126310.1), *FT* (Cor0209610.1 and Cor0024610.1), *SOC1* (Cor0083230.1 and Cor0152090.1), and *TCP* (Cor0043840.1 and Cor0120020.1), suggesting that ChARF3 might regulate the expression of these TFs to coordinate ovule development. Previously, the effects of *FT, SOC1*, and *LFY*, a set of flowering activators, were demonstrated on primordial ovary formation or ovule differentiation and growth (Cheng et al., 2018b). Here, it was demonstrated that this set of flowering activators might be regulated by ChARF3, and new insights into the molecular mechanism of ovule development were obtained.

### Model for ChARF3 Action in the Regulation of Hazel Ovule Development

Based on the results mentioned above, we propose a preliminary model for the ChARF3 transcriptional regulatory framework during ovule development and growth in hazelnut. In the Ov1-vs-Ov2 comparison, downregulated *ChARF3* expression levels might relieve the inhibition of a set of target genes, including *YUC10, LAX5, IAA9/30, SAUR32, PLT2*, and *SOC1*. These target genes regulate auxin biosynthesis and transport, cell expansion, flower development, and fruit initiation (Schrader et al., 2003; Wang et al., 2005; Braybrook et al., 2006; Park et al., 2007; Lee and Lee, 2010; Mähönen et al., 2014; Chettoor and Evans, 2015), suggesting that ChARF3 may coordinate them to regulate ovule formation and early ovule growth. In the Ov2-vs-Ov3 comparison, the upregulation of ChARF3 expression negatively regulated target genes, including *YUC10, PIN7, IAA4, SAUR32, TCP14, AGL21, MFO1, STK, FT*, and *SOC1*. These target genes were involved in auxin biosynthesis and transport, cell division and proliferation, ovule identity, and flower development (Schrader et al., 2003; Gong et al., 2004; Nagpal et al., 2005; Park et al., 2007; Tani et al., 2009; Lee and Lee, 2010; Kieffer et al., 2011; Li et al., 2011; Yu et al., 2014; Chettoor and Evans, 2015; Cheng et al., 2018b; Xiong et al., 2019); inhibition of their expression might be beneficial for rapid ovule growth. Similarly, in the Ov3-vs-Ov4 comparison, upregulated ChARF3 inhibited genes encoding the flowering regulators IAA4, AGL61 and LFY, suggested that ChARF3 contributed to the regulation of ovule maturity through its inhibitory effect on embryo maturation-related gene *IAA4* (Pomares-Viciana et al., 2019) and the floral component development gene *AGL61* (Tekleyohans et al., 2017) and *LFY* (Cheng et al., 2018b). These insights provide a new dimension to ChARF3-mediated gene regulation during ovary initiation and ovule development in hazel.

## Data Availability Statement

The original contributions generated for the study are publicly available. This data can be found here: The RNA-Seq data have been deposited in the NCBI Sequence Read Archive under the accession number PRJNA591492 (https://www.ncbi.nlm.nih.gov/sra/?term=PRJNA591492). The ChIP-Seq data, including raw sequencing data, peak files, and track bigwig files, have been submitted in the NCBI Sequence Read Archive under the accession number PRJNA732731 (https://www.ncbi.nlm.nih.gov/sra/?term=PRJNA732731) and Gene Expression Omnibus (GEO) database under the accession number GSE176170.

## Author Contributions

JL and YC contributed to study conception and design, collection and/or assembly of data. JL, YC, and HW contributed to data analysis and interpretation. HW and JL contributed to writing the manuscript. YS, HW, XZ, and HH prepared samples and performed experiments. HW contributed to the bioinformatics analysis. All authors have read and approved the manuscript.

## Conflict of Interest

The authors declare that the research was conducted in the absence of any commercial or financial relationships that could be construed as a potential conflict of interest.

## Publisher's Note

All claims expressed in this article are solely those of the authors and do not necessarily represent those of their affiliated organizations, or those of the publisher, the editors and the reviewers. Any product that may be evaluated in this article, or claim that may be made by its manufacturer, is not guaranteed or endorsed by the publisher.

## Supplementary Material

Supplementary Figure 1 Specificity of polyclonal antibody against ChARF3.

Supplementary Figure 2 Distribution of the identified motifs in ChARF gene family.

Supplementary Table 1 List of potential ChARF3 target genes at three ovule development stages.

Supplementary Table 2 Motif analysis of ChARF3-binding sites at three ovule development stages.

Supplementary Table 3 Significant enriched KEGG pathways at three ovule development stages in hazel.

Supplementary Table 4 ChARF3 regulated target genes at three ovule development stages.

Supplementary Table 5 List of target genes involved in auxin synthesis, transport, and signaling pathways as well as regulation of ovule development in hazel.
