# Supplementary material for: Genome-Wide Identification of the ARF Gene Family and ARF3 Target Genes Regulating Ovary Initiation in Hazel via ChIP Sequencing
Source: Front Plant Sci. 2021 Aug 10;12:715820. doi: 10.3389/fpls.2021.715820 (PMC8382943; doi:10.3389/fpls.2021.715820)
Supplement: Supplementary Figure 1 — Specificity of polyclonal antibody against ChARF3. [file Data_Sheet_1.docx]

Supplementary Material

# Supplementary Data

## Data Availability Statement

The RNA-seq data with three biological replicates have been deposited in the NCBI Sequence Read Archive (<https://www.ncbi.nlm.nih.gov/sra/?term=PRJNA591492>). The ChIP-Seq data, including raw sequencing data, peak files, and track bigwig files, have been submitted in the NCBI Sequence Read Archive under the accession number PRJNA732731 (https://www.ncbi.nlm.nih.gov/sra/?term= PRJNA732731) and Gene Expression Omnibus (GEO) database under the accession number GSE176170 (<https://www.ncbi.nlm.nih.gov/geo/query/acc.cgi?acc=GSE176170>).

# Supplementary Figures and Tables

## Supplementary Figures


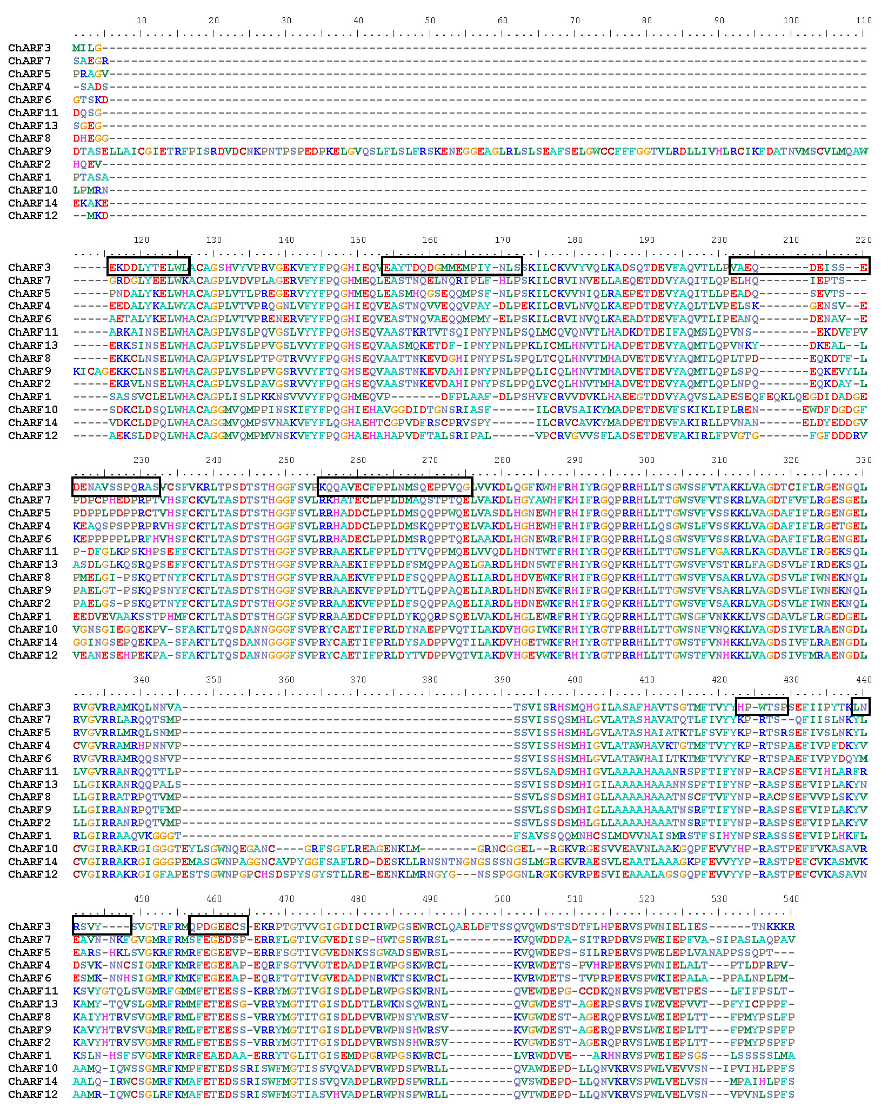


**Supplementary Figure 1.** Specificity of polyclonal antibody against ChARF3. Our gene family bioinformatics analysis with RNA-Seq data revealed 14 specific members of ChARF transcription during hazelnut ovary development. ChARF3 possesses some short peptide sequences (black rectangles) which is specific to homologies of ChARF3 used for developing the antibody.


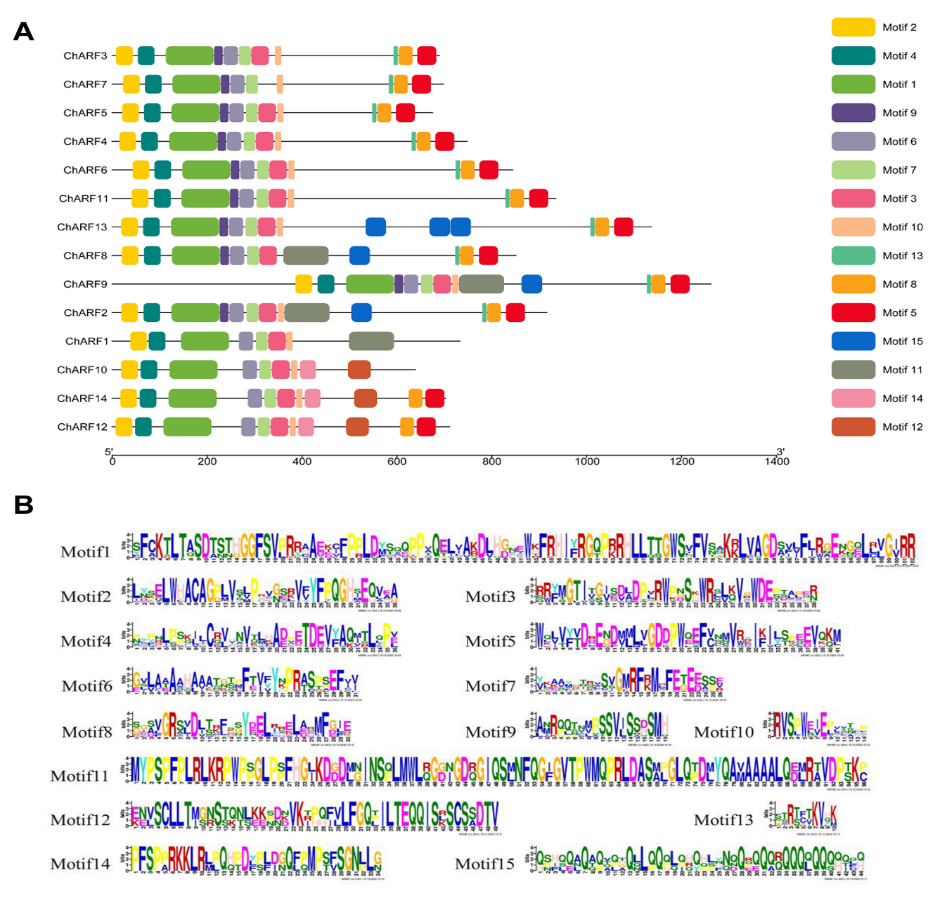


**Supplementary Figure 2.** Distribution of the identified motifs in ChARF gene family. The online software MEME was employed to investigate the motif distributions in all 14 ChARFs, with 15 motifs identified.

## Supplementary Tables

**Supplementary Table 1.** List of potential ChARF3 target genes at three ovule development stages

**Supplementary Table 2.** Motif analysis of ChARF3-binding sites at three ovule development stages

**Supplementary Table 3.** Significant enriched KEGG pathways at three ovule development stages in hazel

**Supplementary Table 4.** ChARF3 regulated target genes at three ovule development stages

**Supplementary Table 5.** List of target genes involved in auxin synthesis, transport, and signaling pathways as well as regulation of ovule development in hazel
